# Taxonomic and Metabolite Diversities of Moss-Associated Actinobacteria from Thailand

**DOI:** 10.3390/metabo12010022

**Published:** 2021-12-27

**Authors:** Chadabhorn Insuk, Pornkanok Pongpamorn, Adrian Forsythe, Atsuko Matsumoto, Satoshi Ōmura, Wasu Pathom-aree, Naowarat Cheeptham, Jianping Xu

**Affiliations:** 1 Department of Biology, McMaster University, Hamilton, ON L8S 4K1, Canada; insukc@mcmaster.ca (C.I.); forsytae@mcmaster.ca (A.F.); 2 National Omics Center, National Science and Technology Development Agency (NSTDA), Pathum Thani 12120, Thailand; pornkanok.pon@nstda.or.th; 3 Kitasato Institute for Life Sciences, Kitasato University, Minato-ku, Tokyo 108-8641, Japan; amatsu@lisci.kitasato-u.ac.jp (A.M.); omuras@insti.kitasato-u.ac.jp (S.Ō.); 4 Research Center of Microbial Diversity and Sustainable Utilization, Faculty of Science, Chiang Mai University, Chiang Mai 50200, Thailand; 5 Department of Biological Sciences, Faculty of Science, Thompson Rivers University, Kamloops, BC V2C 0C8, Canada

**Keywords:** actinobacteria, moss, selective isolation, plant growth promotion, MRSA (methicillin-resistant *Staphylococcus aureus*), white-nose syndrome, VOCs (volatile organic compounds), GC-MS (gas chromatography–mass spectrometry)

## Abstract

Actinobacteria are a group of ecologically important bacteria capable of producing diverse bioactive compounds. However, much remains unknown about the taxonomic and metabolic diversities of actinobacteria from many geographic regions and ecological niches. In this study, we report the isolation of actinobacteria from moss and moss-associated rhizosphere soils in Thailand. Among the 89 isolates analyzed for their bioactivities, 86 strains produced indole-3-acetic acid (IAA, ranging from 0.04 to 59.12 mg/L); 42 strains produced hydroxamate type of siderophore; 35 strains produced catecholate type of siderophore; 21 strains solubilized tricalcium phosphate; and many strains exhibited antagonistic activities against one to several of the seven selected plant, animal, and human pathogens. Overall, actinobacteria from the rhizosphere soil of mosses showed greater abilities to produce IAA and siderophores and to solubilize tricalcium phosphate than those from mosses. Among these 89 isolates, 37 were analyzed for their 16S rRNA gene sequences, which revealed their diverse phylogenetic distributions among seven genera, *Streptomyces*, *Micromonospora*, *Nocardia*, *Actinoplanes*, *Saccharothrix*, *Streptosporangium*, and *Cryptosporangium*. Furthermore, gas chromatography-mass spectrometry analyses of ethyl acetate crude extracts of three selected isolates with inhibitory effects against a methicillin-resistant *Staphylococcus aureus* strain revealed diverse metabolites with known antimicrobial activities. Together, our results demonstrate that actinobacteria from mosses in Thailand are taxonomically diverse and capable of producing a range of metabolites with plant-growth-promoting and microbial pathogen-inhibiting potentials.

## 1. Introduction

Plant-associated microorganisms have shown an outstanding ability to produce compounds of high therapeutic value [1]. Among all bacterial taxa associated with plants, *Actinobacteria*, a phylum of Gram-positive, high GC-content bacteria, are among the most prolific producers of secondary metabolites, especially antibiotics. *Actinobacteria* are common components of endophytic microbiota of medicinal plants [2,3] and have been found in the rhizosphere of numerous plants, including crops, such as wheat [4], yam [5], and pea [6]. These plant-associated actinobacteria can employ several mechanisms to influence plant growth, such as enhancing nitrogen fixation, phosphate solubilization, iron acquisition through secreting siderophores, and production of phytohormones [7]. They can also protect plants by inhibiting plant pathogens [8], enhancing abiotic stress tolerance [9,10], and maintaining overall soil health [11]. However, most of our understandings regarding plant-associated actinobacteria have come from studies in higher plants. The investigation of actinobacteria from lower plants, such as bryophytes (including mosses), and their beneficial traits are still rare. Recently, a member of the actinobacteria genus *Micromonospora* was reported from bryophytes, which showed plant growth-promoting activity in vitro and in planta [12]. Such a result suggests a potentially important role of actinobacteria in mosses.

Mosses are a group of early-diverging land plants that naturally exhibit high tolerance to extreme desiccation and can return to their normal metabolism very rapidly after rehydration. Microorganisms associated with mosses likely experience similar stress conditions [13]. Ecologically, mosses are found in a variety of ecosystems, such as on the ground, tree trunks, or even human-made concrete surfaces. They play important roles in food web dynamics, carbon and nutrient cycling, and soil temperature and moisture regulations [14]. Previous research has shown that moss-associated microbes are capable of enhancing the growth of the moss *Physcomitrium sphaericum* during its acclimatization from the laboratory environment to natural field soil [12], fix N_2_ in the air [15], and contribute to the process of biomass decomposition [16].

In this study, we hypothesized that actinobacteria from mosses from Thailand are diverse and capable of producing both plant growth-promoting metabolites as well as antibiotics effective against diverse pathogens. To test their potential productions of antimicrobial compounds, we selected several representative pathogens of global significance to plants, animals, and humans. For plant pathogens, we choose *Pseudomonas syringae* pv. *syringae* and *Pseudomonas aeruginosa* for testing. *Pseudomonas syringae* is a globally distributed plant pathogenic bacterium that can cause diseases in monocotyledon, herbaceous dicotyledon, and woody dicotyledon plants [17,18,19,20]. There are more than 60 pathovars of *P. syringae*, but the most polyphagous one is *P. syringae* pv. s*yringae* with its wide host range and virulence factors [21]. *Pseudomonas aeruginosa* is an opportunistic pathogen of diverse plants and animals, including humans [22,23,24,25]. For the animal pathogen, we choose *Pseudogymnoascus destructans*, a psychrophilic fungal pathogen and the causative agent of white-nose syndrome (WNS) that has caused significant mortality to hibernating bats in North America [26,27]. For human pathogens, we selected four strains representing three common pathogens for testing: two strains belong to the Gram-positive *Staphylococcus aureus* and one strain each of the Gram-negative *Escherichia coli* and the opportunistic yeast pathogen *Candida albicans*. The *E. coli* strain was resistant to multiple antibiotics, and one of the two strains of *S. aureus* was resistant to methicillin. We are specifically interested in whether actinobacteria from mosses are capable of inhibiting the growth of diverse human pathogens, including drug-resistant bacterial pathogens. If such activities are found, the actinobacteria from mosses could represent a potential source for novel antibiotics to help combat drug-resistant infections [28].

To achieve our objectives and test our hypothesis, we isolated cultivable actinobacteria associated with various moss species in northern Thailand. The potential taxonomic affiliations of selected isolates were investigated based on their 16S rRNA gene sequences. Their abilities to produce plant-growth promoters and to inhibit the growth of plant pathogens, human pathogens, and bat pathogens were also determined. Studying actinobacteria associated with mosses and their ability to produce bioactive compounds can help us not only understand the interactions between actinobacteria and mosses but also discover potential new bioactive compounds to broaden drug discovery.

## 2. Results

### 2.1. Isolation of Actinobacteria from Mosses

The total samples used in this study belonged to nine moss species, consisting of *Pogonatum microstomum* (R. Br. ex Schwägr.) Brid. (No. 3), *Hypnum sp.* (No. 6), *Thuidium cymbifolium* (Dozy & Molk.) Dozy & Molk. (No. 11), *Plagiomnium maximoviczii* (Lindb.) T.J. Kop (No. 13), *Brachythecium buchananii* (Hook.) A. Jaeger (No. 32), *Didymodon maschalogena* (Renauld & Cardot) Broth. (No. 33), *Macrothamnium submacrocarpum* (A. Jaeger ex Renauld & Cardot) M. Fleisch. (No. 49), *Ceratodon purpureus* (Hedw.) Brid. (No. 52), and *Bryum recurvulum* Mitt. (No. 54). The numbers in the parenthesis correspond to the codes of moss samples in the herbarium. These numbers are also used as part of the name for the bacterial isolates from those moss samples. After species identification, the moss samples were dried and kept at Chiang Mai University Herbarium (Herbarium code: CMUB). The geographic coordinates for each of the moss samples are listed in Table 1.

Two commonly used media (V8 juice agar and water proline agar, both supplemented with antibiotics) for isolating actinobacteria and two pre-treatment temperatures (25 and 60 °C) were used in this study to isolate actinobacteria. A total of 196 isolates of actinobacteria were obtained from the nine moss species (Table 1). A higher number of isolates was obtained from V8 juice agar (108 isolates, 55.1%) compared to water proline agar (Table 1). Actinobacterial counts inside individual moss plants ranged from 0 to 3 × 10^5^ CFU/g (Table 1). A slightly lower count was obtained from the rhizosphere soil samples (0–5.9 × 10^4^ CFU/g, Table 1). The average actinobacterial count was 8.58 × 10^4^ CFU/g inside mosses and 1.83 × 10^4^ CFU/g in the rhizosphere soil of mosses. The highest number of isolates was obtained from moss species *B. buchananii* (75 isolates, 38.27%), followed by *P. microstomum* (51 isolates, 26.02%), *Hypnum* sp. (29 isolates, 14.8%), and *B. recurvulum* (25 isolates, 12.76%) (Table 1). Comparing the efficiency of the two isolation methods, the first method showed abundant growth of fast-growing bacteria that were not actinobacteria. The drying process at 60 °C in the second method worked well at eliminating fast-growing high-temperature-sensitive bacteria and yielded up to 10^5^ CFU/g of actinobacteria. Based on their colony morphology on Seino’s and Waksman’s agar slants, these 196 isolates of actinobacteria were grouped and reduced to 89 isolates for the remaining analyses. Each of the 89 isolates had a distinct colony morphology on Seino’s and/or Waksman’s agar slants.

### 2.2. Testing for Plant Growth Promoting Potential

In this study, we examined the production of three known types of plant-growth-promoting substances by the actinobacteria isolated from mosses and moss-associated soil in Thailand. The three types of substances are indole-3-acetic acid (IAA), siderophores, and tricalcium phosphate solubilizers.

**Figure 1:**
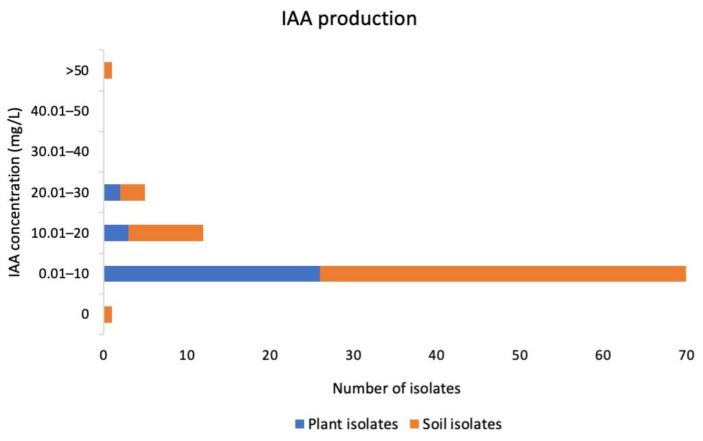
IAA production by actinobacteria from mosses.

Among the 89 analyzed isolates of actinobacteria, 86 (96.63%) produced detectable levels of indole-3-Acetic Acid (IAA) in vitro, ranging from 0.04 (± 0.08) to 59.12 (± 1.99) mg/L in medium with 2 mg/mL L-tryptophan supplementation. Most actinobacteria (76.40%) from mosses produced IAA in the range of 0.01–10 mg/L (Figure 1). Isolate S3-21 produced the highest IAA at 59.12 mg/L. On average, rhizosphere actinobacteria produced slightly more IAA (7.37 ± 1.66 mg/L) than endophytic actinobacteria (6.51 ± 1.31 mg/L).

**Figure 2:**
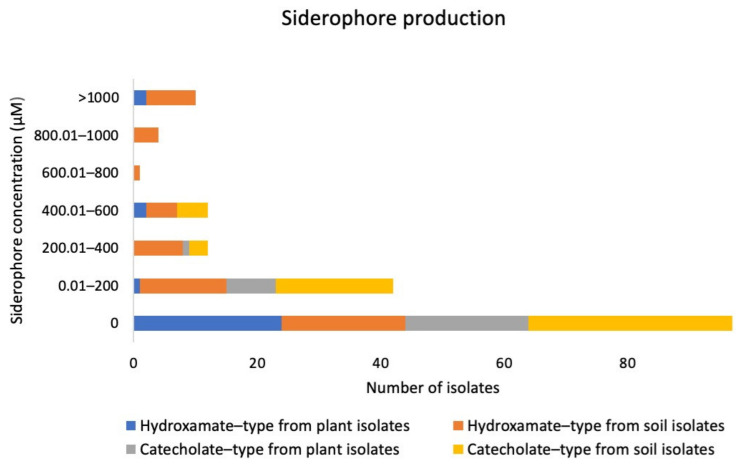
Siderophore production of actinobacteria associated with mosses.

A subset of the actinobacteria produced both hydroxamate and catecholate types of siderophores (Figure 2), with 42 isolates producing the hydroxamate type of siderophore (47.19%) and 35 isolates producing the catecholate type of siderophore (39.33%). For hydroxamate siderophore, isolate S32-74 produced the highest amount, at 1949 µM. For catecholate siderophore, isolate S32-27 produced the highest amount, at 547 µM (Figure 2). On average, rhizosphere isolates produced a higher concentration of siderophore than endophytic isolates. The average catecholate siderophore from rhizosphere actinobacteria was 69.36 ± 13.90 µM and from endophytic actinobacteria was 24.64 ± 6.15 µM. The average hydroxamate siderophores from rhizosphere actinobacteria and endophytic actinobacteria were 358.43 ± 73.61 and 107.33 ± 31.50 µM, respectively. Isolate S32-74 produced the highest hydroxamate siderophore at 1949.17 ± 80.09 µM and isolates S32-27, S32-29, S32-5, and S32-74 produced the highest catecholate siderophore at 489.82 ± 30.84–547.02 ± 138.14 µM. These differences are statistically significant at *p* < 0.05 based on one-way ANOVA and Tukey HSD test using the program SPSS.

Tests of phosphate solubilization ability showed that 20 isolates (22.47%) exhibited clear zones on PVK agar, indicating their ability to solubilize tricalcium phosphate. Overall, actinobacteria isolated from moss-associated soil showed larger clear zones for phosphate solubilization than actinobacteria obtained from inside mosses. Soil isolate S6-28 produced the largest clear zone on PVK agar at 1.75 ± 0.12 cm (Table 2).

### 2.3. Evaluation of Antimicrobial Activity

**Figure 3:**
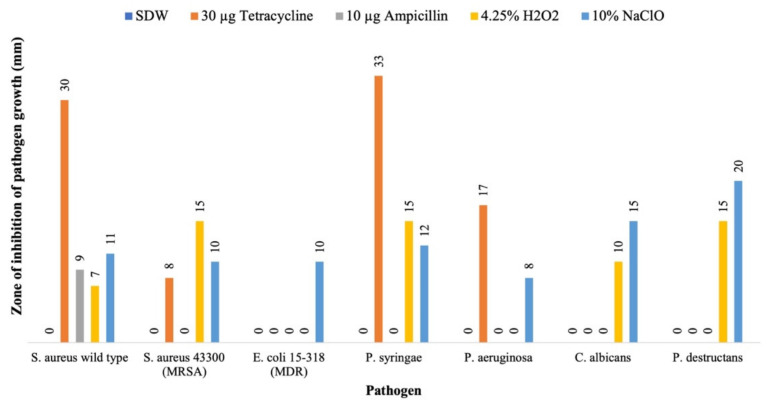
Susceptibilities of the seven microbial pathogens to two known antibiotics and two chemical agents used in this study.

We tested the antimicrobial activities of all 89 actinobacteria isolates from mosses against the seven pathogenic microbial strains. For comparative purposes, we also tested the susceptibilities of these seven pathogens to known antibiotics and/or disinfectants. Our results showed that *S. aureus* ATCC 8923 was susceptible to ampicillin and tetracycline, and the two tested disinfectants H_2_O_2_ and NaClO. However, strain MRSA 43300 was resistant to ampicillin. Strain *E. coli* 15-318 was resistant to almost all tested drugs and chemicals, except to 10% NaClO (10 mm clear zone). Strain *P. aeruginosa* 13 was susceptible to 10% NaClO (8 mm) and tetracycline (17 mm) but not susceptible to 4.25% H_2_O_2_. The *P. syringae* pv. *syringae* strain 19874 was susceptible to 10% NaClO (12 mm), 4.25% H_2_O_2_ (15 mm), and tetracycline (33 mm). For the *P. destructans* strain US15, 4.25% H_2_O_2_ and 10% NaClO showed full inhibitions with the zones of inhibition of 15 and 20 mm, respectively (Figure 3). However, because each of the 35 treatments (sterile water + four chemical agents × seven pathogens) was tested only once, we were unable to determine the statistical significance of the observed differences among treatments.

**Figure 4:**
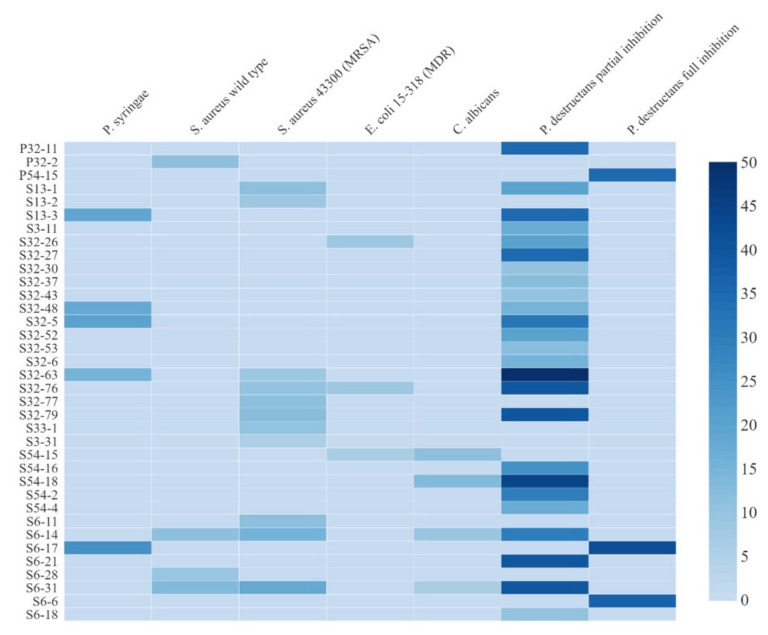
Heat map representing antimicrobial activity of actinobacteria from mosses against two drug-resistant bacteria and two fungal pathogens. The legend on the right size indicates the diameter of the zone of inhibition (in mm).

**Figure 5:**
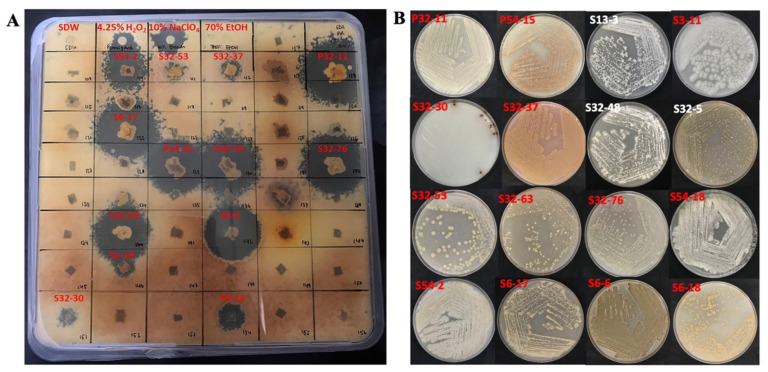
Representative strains that showed inhibition activity against *P. destructans.* (**A**) Anti-*P. destructans* activity of actinobacteria from mosses. (**B**) Colony morphological diversity of representative actinobacteria that showed activities against diverse pathogens. Isolates were grown on an ISP3 medium for 2 weeks at 28 °C. Letters in red indicate isolates with anti-*P. destructans* activity that also showed in (**A**).

Among the 89 actinobacteria isolates, 16 showed an inhibitory ability against both *P. syringae* pv. *syringae* and *P. destructans*. However, none of the 89 actinobacteria showed inhibition against *P. aeruginosa*. Several isolates showed a broad range of inhibitions, such as isolates S32-63 and S6-14. Isolate S32-63 inhibited the Gram-positive MRSA strain, the Gram-negative *P. syringae* pv. *syringae*, and the fungus *P. destructans*. Isolate S6-14 inhibited strain *S. aureus* ATCC 8923, the yeast *C. albicans*, and *P. destructans*. Several isolates, such as P32-2, P32-11, and P54-15, inhibited only one pathogen each (Figure 4). Interestingly, isolates P54-15, S6-17, and S6-6 completely inhibited the growth of *P. destructans* on bioassay plates (Figure 5A). Interestingly, 70% EtOH did not inhibit the growth of *P. destructans* (Figure 5A). The morphology of several isolates showing strong antimicrobial activities against the seven pathogens are displayed in Figure 5B.

### 2.4. 16S rRNA Analysis of Actinobacteria from Mosses

**Figure 6:**
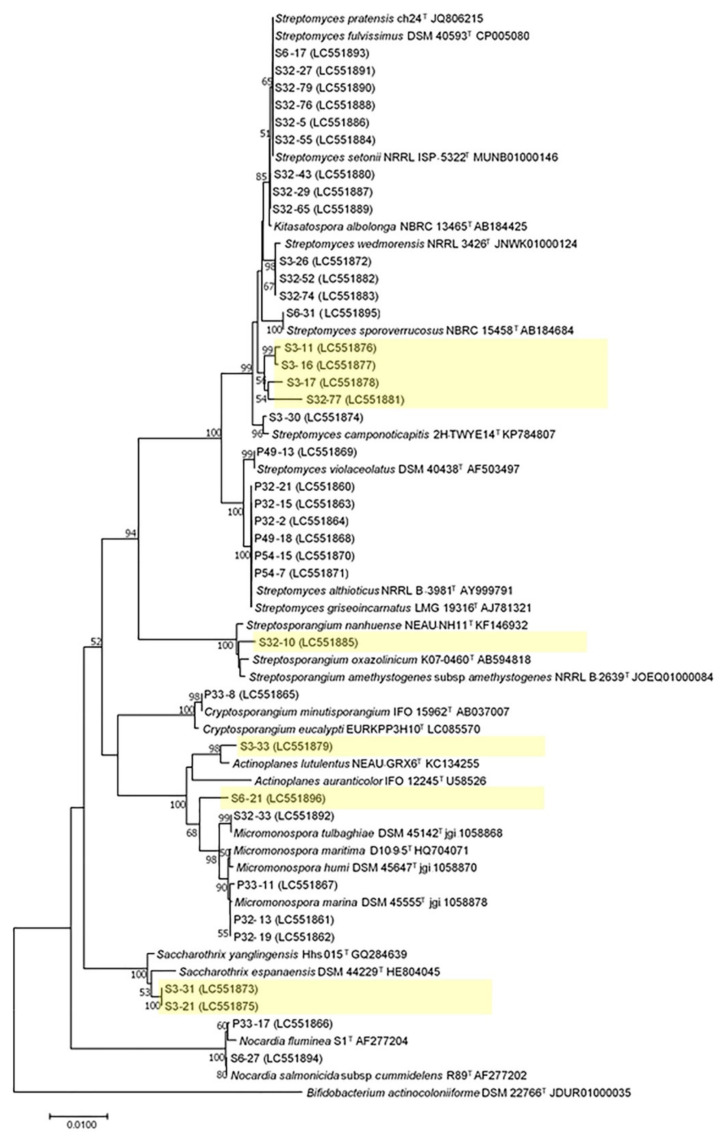
Phylogenetic tree of actinobacteria from mosses. The tree was inferred using the Neighbor-Joining method [29]. The percentage of replicate trees in which the associated taxa clustered together in the bootstrap test (1000 replicates) are shown next to the branches. The values less than 50 were removed. The evolutionary distances were computed using the Maximum Composite Likelihood method [30]. Evolutionary analyses were conducted in MEGA7. The five highlighted clades in yellow likely belong to seven new species.

Among the 89 isolates, 37 strains were selected for 16S rRNA sequencing based on their morphological characteristics on ISP2 and ISP3 agar and their antimicrobial activities. These isolates were chosen based on their morphological characteristics on ISP2 and ISP3 agar and their bioactivities. The majority of these 37 actinobacteria belonged to *Streptomyces* (25 isolates, 67.57%), which were followed by *Micromonospora* (4 isolates, 10.81%), *Nocardia* (2 isolates, 5.41%), *Actinoplanes* (2 isolates, 5.41%), *Saccharothrix* (2 isolates, 5.41%), *Streptosporangium* (1 isolate, 2.7%), and *Cryptosporangium* (1 isolate, 2.7%). All these isolates shared between 98.57% and 100% 16S rRNA gene sequence similarity with their nearest known species (Table 3). Phylogenetic analysis confirmed the assignments of all isolates to corresponding genera, in agreement with the BLAST results (Figure 6). Based on the sequence analysis, these 37 isolates likely represent 19 species, with several of them representing potential new species (highlighted in yellow boxes). These seven putative new species were proposed because their 16S rRNA sequences showed greater divergence with their closest known species than the sequence differences between the known sister species within their respective genera (Figure 6).

### 2.5. GC-MS Results

Three strains (*S. fulvissimus* strain S32-76, *S. dioscori* strain S32-77, and *S. setonii* strain S32-79) that showed significant antimicrobial activities were chosen for analysis of their metabolite profiles. We compared the results of the GC-MS of each strain with the GC-MS profiles of the pure sterilized liquid medium that underwent the same treatments to remove the background noises and to confirm that the compounds were released from actinobacterial cells into the liquid media. Solvent extraction was performed to obtain volatile organic compounds (VOCs) in the liquid media. Then the crude extract was subjected to GC-MS analysis. To obtain a more comprehensive profile of metabolites for each isolate, the crude extract of each isolate was analyzed using two approaches: one without derivatization and the second with derivatization. The process of derivatization allows certain types of metabolites, such as acids and bases, that are otherwise difficult to volatilize by themselves to become volatile with the help of a silylating reagent.

As expected, derivatization had an effect on the detected compounds (Table 4). The major class of compounds before derivatization were alkane (46.75%), alcohol (12.99%), and ketone (3.90%). After derivatization, the major compounds were alkanes (15.29%), carboxylic acid (10.59%), sugar (8.24%), and alcohol (7.06%). The details of individual compounds for each strain are presented in Supplementary Table S1. Interestingly, tryptophol was detected from S32-79 before derivatization but not after derivatization. In contrast, maltol was found in the crude extract of strain S32-76 after derivatization but not before. Tris(2,4-di-tert-butylphenyl) phosphate was found with the highest peak area of all isolates before derivatization but disappeared after derivatization.

For each of the three isolates, the most prominent peaks between pre- and post-derivatization were different. Specifically, prominent peaks of S32-76 before derivatization were dihydroergotamine and after derivatization was galactose oxime, with peak areas 0.83% and 2.07%. They exhibited retention times corresponding to 27.094 and 21.959, respectively. Prominent peaks of S32-77 before and after derivatization were Tris(2,4-di-tert-butylphenyl) phosphate and pentadecanoic acid, with peak areas 18.13% and 0.91% and retention times of 50.947 and 22.19, respectively. Prominent peaks of strain S32-79 before and after derivatization were Tris(2,4-di-tert-butylphenyl) phosphate and hymexazole, tert-butyldimethylsilyl ether, with peak areas 10.59% and 1.21% and retention times of 50.979 and 11.62, respectively. However, it should be noted that peak areas cannot be used to directly calculate the amount of individual compounds. This is because the peak area is not only influenced by the amount of each compound in the extract but also by the properties of the compounds, especially their volatility and susceptibility to ionization. In addition, we observed some prominent unknown peaks that did not match any known compound in the database. For example, in strain S32-79, there are several unknown compounds, such as ones at retention times 21.006 and 22.791. Derivatization increased the number of unidentified compounds; for example, in S32-79, GC-MS detected 22 unidentified compounds in pre-derivatization and 128 unidentified compounds in post-derivatization.

The GC-MS revealed that the three isolates were able to produce diverse putative bioactive compounds in vitro (Supplementary Figure S1). Some VOCs were found in common among the three isolates, including hexadecane and 3-methyl-octadecane. Among the three isolates, the highest number of VOCs before derivatization was detected in strain S32-76 with 36 VOCs. After derivatization, strain S32-79 showed the highest number of VOCs with 33 VOCs. The details of the GC-MS profile for each of the three isolates before and after derivatization are shown in Supplementary Table S1. References [31,32,33,34,35,36,37,38,39,40,41,42,43,44,45,46,47,48,49,50,51,52,53,54,55,56,57,58,59,60,61,62,63,64,65,66,67,68,69,70,71,72,73,74,75,76,77,78] are included in Supplementary Table S1 and they describe the features of the compounds we identified through GC-MS.

Before derivatization, VOCs commonly shared between *S. fulvissimus* S32-76 and *S. dioscori* S32-77 were dihydroergotamine, octacosanol, pinostrobin, octadecylcyclohexane, 10,11-dihydrofarnesol, 5-methylheneicosane, and 1-iodo-octacosane. The most commonly shared VOCs between *S. dioscori* S32-77 and *S. setonii* S32-79 were 3-methyl-octadecane and hexadecane. Furthermore, the commonly shared VOCs between *S. fulvissimus* S32-76 and *S. setonii* S32-79 were phenylethyl alcohol, 1,1’-(1,3-phenylene) bisethenone, 3,6-diisopropyl-2,5-dioxomorpholine, and pentacosane. However, a large number of VOCs were not shared among the three strains, including (i) cyclotetradecane, 3-methyl-heptadecane, and humulenol-II (found only in S32-76); (ii) 1-octadecanol methyl ether, crotonic acid menthyl ester, 2-dodecen-1-yl(-)succinic anhydride (found only in S32-77); and maltol, succinimide, and tryptophol (found only in S32-79).

After derivatization, the VOCs shared among all three isolates were p-benzoquinone, myristic acid, 5-methyl-heneicosane, pyrrole-2-carboxylic acid, erythritol, and 4-hydroxybenzyl alcohol. The VOCs found in both *S. fulvissimus* S32-76 and *S. dioscori* S32-77 include 1-phenyl-1,2-ethanediol, melamine N-(trimethylsilyl), hexadecane, and β-gentiobiose. The VOCs shared between *S. dioscori* S32-77 and *S. setonii* S32-79 were pyrrole-2-carboxylic acid, arabinofuranose; pentadecanoic acid, and 1-monopalmitin. The VOCs shared between *S. fulvissimus* S32-76 and *S. setonii* S32-79 were phenylethyl alcohol, p-toluic acid, anthranilic acid, (1E)-d-Glucose-2,3,4,5,6-pentakis-O-(trimethylsilyl)-o-methyloxyme, glucose, and tetracontane. Some VOCs after derivatization were found only in one of the three strains, e.g., urea, xylitol, and tryptophol in strain S32-76; benzenebutanoic acid, crotonic acid-menthyl ester in strain S32-77; and pyruvic acid and maltol in strain S32-79.

**Figure 7:**
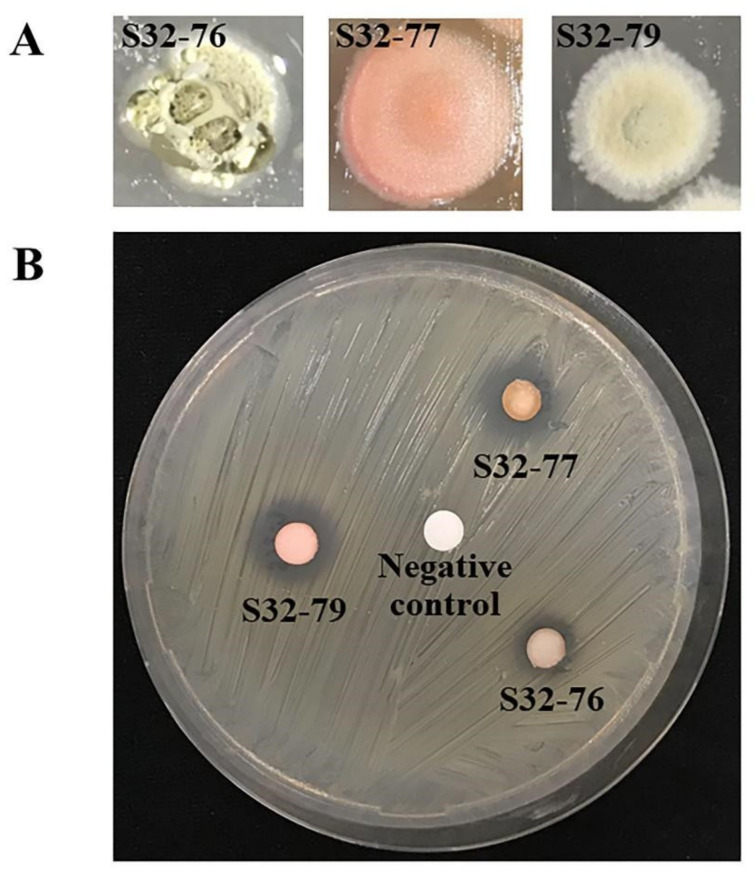
Colonies of three selected *Streptomyces* isolates (strains S32-76, S32-77, and S32-79) (**A**); and their activities against MRSA in their ethyl acetate extracts of cell culture supernatant (**B**). In (**A**), colony morphology was observed on an ISP3 medium after seven days of incubation at 28 °C under stereomicroscope. In (**B**), three paper disks (6 mm in diameter) loaded with 40 µL of crude extracts were plated on nutrient agar overlayed by MRSA. The center was a negative control disk loaded with 40 μL of absolute ethanol. All discs were completely dried before placing on the plate.

GC-MS detected several bioactive compounds with reported bioactivity in promoting plant growth, including 2,3-dihydroxybenzoic acid, a catecholate siderophore with antimicrobial activity, and L-tryptophan, a substrate for IAA production, in the crude extract of S32-77. The ethyl acetate extracts of strains S32-76, S32-77, and S32-79 were also tested against MRSA. These extracts inhibited the growth of MRSA with the sizes of clear zones ranging from 10 to 14 mm (Table 5). These results confirm the bioactivity of actinobacterial extract injected into GC-MS. The activity of MRSA inhibition and colony of three potential isolates were represented in Figure 7.

## 3. Discussion

In this study, we surveyed the diversity of actinobacteria from representative mosses in northern Thailand and investigated their potential bioactive repertoires. Our surveys revealed that actinobacteria were common both inside mosses (with a mean of 8.58 × 10^4^ CFU/g) and in the rhizosphere soil of mosses (mean of 1.83 × 10^4^ CFU/g). An early study from Japan [79] reported the mean density of actinobacteria in the rhizosphere of diverse vascular plants was around 10^7^ CFU g^−1^ (dried sample) and a range of 10^3^–10^5^ CFU g^−1^ (dried sample) from the tissues of plants. Our estimated low density of actinobacteria in rhizosphere soil of mosses might be due to the differences in soil characteristics and geographic locations. For example, the optimum soil pH of bryophytes (including mosses) is slightly acidic, ranges from 4.5 to 7.2 [80], and is not conducive to actinobacteria growth. Indeed, previous studies showed that the relative abundance of actinobacteria decreased with decreasing pH [81]. Similarly, in waterlogged, anaerobic soils and acidic soils, the numbers of actinobacteria are typically low (10^2^–10^3^ g^−1^ dry weight soil), lower than what we found here [82].

The preheating step for soil and moss samples eliminated fast-growing microbes and enhanced the recovery of the slow-growing actinobacteria from mosses up to 10^5^ CFU/g. Heat pre-treatment has been frequently used for removing non-actinobacteria microbes from soil samples to help obtain a higher amount of rare actinobacteria [83]. V8 juice agar, which has been widely used for fungal isolation, was also conducive for isolating actinobacteria when supplemented with Benomyl, a fungicide. Water proline agar (WPA) is generally preferred by *Streptomyces* [84]. However, we found that WPA resulted in a similar growth density of actinobacteria as V8 juice agar. Humic acid vitamin agar (HVA), tap water yeast extract agar (TWYA), and starch casein agar (SCA) are the other frequently used isolation media for endophytic actinobacteria [85]. Additional attempts using these three types of media (HVA, TWYA, and SCA) may allow us to obtain novel actinobacteria species and strains. For plant samples, surface sterilization of plant parts before plating on isolation media are often sufficient to remove epiphytic microbes and enhance the efficiency for isolating actinobacteria from the plant endosphere [85,86]. Overall, our results showed that the protocols used in our study were effective at isolating actinobacteria from mosses and moss-associated soils.

Actinobacteria are known to produce plant hormones, such as auxins, gibberellin, and cytokinin. In this study, *Saccharothrix yanglingensis* Hhs.015^T^ S3-21 produced the highest IAA at 59.12 ± 1.99 mg/L on medium supplemented with 2 mg/mL L-tryptophan. Overall, IAA production of actinobacteria from mosses was similar or lower than those of actinobacteria from other plants. For example, *Streptomyces* sp. strain DBT204, an endophytic actinobacterium of tomato *Solanum lycopersicum* produced 46.3 mg/L IAA under conditions identical to what we used here, lower than our highest IAA-producing strain S3-21 but higher than most of our other 88 strains [87]. Thus far, the highest IAA-producing actinobacteria, at 222.75 mg/L, was isolated from mandarin orange [88].

Siderophores are low-molecular-weight chelating agents (200–2000 Da) that have various chemical structures belonging to at least 500 different compounds [89]. Siderophores can also act as signaling molecules. In mammalian hosts, siderophores are required for full virulence of pathogens during infection [90,91]. As iron is a macronutrient in plants, the production of siderophores by actinobacteria can increase iron acquisition and uptake from the environment, thus enhancing the availability of iron to plant roots in the form of bacterial siderophore-iron complex, or phytosiderophore-iron [1]. The production of siderophore by plant-beneficial actinobacteria in biocontrol applications can limit the access of iron to phytopathogenic bacteria. Indeed, 86.8% of actinobacteria isolated from tomato plants could produce siderophores, and they have been shown to contribute to tomato growth. Actinomycetes isolated from eaglewood could produce hydroxamate-type siderophore ranging between 3.21 ± 0.12 and 39.30 ± 0.40 μg/mL and catechols-type at 4.12 ± 0.90 μg/mL [92]. Prior to our study, the highest hydroxamate siderophore production by Actinobacteria from bryophytes was at 992.50 ± 50.76 mM (1.51 μg/mL), and the highest catecholate siderophore was at 484.47 ± 27.91 mM (3.14 μg/mL) [12]. In our study, high percentages of actinobacteria from mosses produced hydroxamate and catecholate siderophores. The highest siderophore productions of our actinobacteria from mosses were 1949.17 ± 80.09 (2.97 μg/mL) and 547.02 ± 138.14 µM (3.55 μg/mL) for hydroxamate and catecholate siderophores, respectively, higher than those reported previously. At present, the reasons for the differences in the amount of IAA and siderophore productions among actinobacteria from mosses and between mosses and other plants are not known.

Phosphate is an essential nutrient in plant growth and development. Phosphorus-solubilizing microorganisms (PSMs) can release organic acid or H^+^ ions to help plants access the reservoir of non-labile phosphorus and release it from its recalcitrant forms [93]. Previous studies have shown that some actinobacteria can solubilize phosphate through different mechanisms. For example, using a synthetic minimal medium with rock phosphate as a sole carbon source, several actinobacteria in genera *Streptomyces* and *Micromonospora* converted rock phosphate into soluble phosphate by the production of siderophore, not organic acids [94]. The proportion of actinobacteria that could solubilize phosphate in our sample was similar to that reported earlier [95] that showed 25.9% of actinobacteria from *Rhynchotoechum ellipticum* endospheric tissues could solubilize phosphate under in vitro conditions determined by a clear zone on PVK agar. Actinobacteria in the genus *Streptomyces* and *Acinetobacter* [96] are known for their ability to solubilize phosphate. In this study, we discovered that actinobacteria belonging to three genera *Nocardia*, *Streptomyces*, and *Saccharothrix* were capable of solubilizing phosphate on PVK agar. Previous studies have shown that inoculating plants with plant-growth-promoting rhizobacteria (PGPR) or treating plants with microbe-to-plant signal compounds could stimulate plant growth and improve crop tolerance against abiotic stresses [93]. In addition, our previous study showed that inoculation of plant-growth-promoting actinobacteria to moss gametophytes increased fresh weight, dry weight, carotenoid content, and capsule production in mosses, and helped mosses acclimatize to outdoor field soil conditions [12]. Indeed, actinobacteria, as well as microbes in other genera, such as *Bacillus*, *Pseudomonas*, and *Lactobacillus*, have been used commercially to enhance plant productions [93]. We believe that the actinobacteria obtained here could also enhance the growth and reproduction of mosses. However, further experimental investigations are needed to confirm their plant growth-enhancing roles.

The bioassay of actinobacteria from mosses against *P. aeruginosa* 13 and *P. syringae* pv. *syringae* 19874, MRSA 43300, *S. aureus* ATCC 8923, *E. coli* 15-318, *C. albicans* 012 and *P. destructans* strain US15 showed different results. Five and thirteen actinobacteria inhibited *P. syringae* pv. *syringae* and *P. destructans*, respectively. Isolate S6-17 was closely related to *Streptomyces setonii* and S32-5 was closely related to *Streptomyces fulvissimus*, both inhibited *P. syringae* pv. *syringae*. There have been successful examples of using actinomycetes or their active compounds to treat *P. syringae* infections. For example, Kasugamycin is an aminoglycoside antibiotic isolated from *Streptomyces kasugaensis* that is approved for controlling *P. syringae* pv. *actinidiae*, the bacteria causing kiwifruit canker [97]. Similarly, Actinomycetes from hypersaline soil in Turkey inhibited *P. syringae* pv. *tomato* in vitro [98] and Actinomycetes isolated from Antarctica showed inhibition on phytopathogenic bacteria, *P. syringae* pv. *syringae* and *P. syringae* pv. *tabaci* [99].

Interestingly, *Streptomyces* from this study, could inhibit *P. destructans* growth effectively. Isolate P54-15 (closely related to *Streptomyces althioticus*) and S6-17 (closely related to *Streptomyces setonii*) showed full inhibition against *P. destructans*. S3-11 (closely related to *Streptomyces rhizosphaerihabitans*) and S32-76 (closely related to *Streptomyces fulvissimus*) showed partial inhibition against *P. destructans.* The results here are consistent with an earlier study [100], showing that the most effective bacterial antagonists against *P. destructans* were from the genera *Bacillus*, *Pantoea*, *Streptomyces*, *Pseudomonas*, *Rahnella*, *Arthrobacter*, and *Sphingobium*. The volatile compounds produced by antagonists, such as 2-methyl-1-propanol, 2-methyl-1-butanol, propanoic acid, and 1-pentanol, could inhibit the growth of *P. destructans* [100]. The mechanism for the observed antifungal antagonists was likely due to the secretion of antifungal compounds and/or hydrolytic enzymes, such as chitinase, β,3 glucanase, chitosanase, and proteases [84].

In this study, we found that isolates from the rhizosphere soil of mosses showed a greater ability to inhibit the pathogens than those of endophytic isolates from mosses. Indeed, populations of actinobacteria inside and outside mosses were found to have distinct characteristics in both their species distributions and putative functions. Based on morphological characteristics and molecular identifications, out of the seven genera, two (*Cryptosporangium* and *Nocardia)* were only isolated from inside mosses, not from their rhizosphere soil. In contrast, three genera (*Saccharothrix*, *Actinoplanes*, and *Streptosporangium)* were only isolated from soil samples but not inside mosses. Overall, in our samples, the species richness in the rhizosphere soil was higher than the moss’s interior. Our results also suggest several potentially new species (shown in yellow highlighted in Figure 6) in the genera *Actinoplanes*, *Saccharothrix*, and *Streptomyces*. Indeed, a novel species of actinobacterium *Streptomyces bryophytorum* was recently isolated from an unidentified moss [101]. Among the seven genera, *Cryptosporangium* spp. has also been isolated from plant litter [102,103] or as endophytes of plants [104]. Due to the close phylogenetic relationships among known species and between many of our samples and those known species, the final taxonomic placements of most of our isolates, especially the yellow-highlighted ones, such as S3-21 and S3-31, in Figure 6, await further investigation using either multilocus sequence typing or whole-genome sequencing. Soil is a dominant niche of actinobacteria, especially for genera *Sacchrothrix* and *Actinoplanes* [105,106,107,108,109,110]. A previous metagenomic analysis of endophytic bacteria of the xerophilous moss *Grimmia montana* revealed the bacterial community inside this moss was composed of Proteobacteria, Firmicutes, Actinobacteria, and *Cytophaga/Flexibacter/Bacteroids* [13]. Overall, metagenomics-based studies of endophytic actinobacteria revealed that *Streptomyces* sp. was the most abundant genus [1,3], similar to what we found in this study.

Previous studies have shown that polyketide (PK) and non-ribosomal peptide (NRP) natural products from *Streptomyces* contain notable antimicrobial and anticancer activities (e.g., [111]). Type III PKSs were originally believed to be plant-specific, but it was later found in *S. griseus* in 1999 [112]. With the use of genetic screening, traditional Chinese herbs were found to contain endophytes capable of synthesizing bioactive compounds through the non-ribosomal peptide synthetase (NRPS) and polyketide synthase (PKS) genes [113]. In addition to their antimicrobial activities, a variety of siderophores are encoded from NRPS genes [112,113]. Whole-genome sequencing of the isolates with strong antimicrobial activities will help reveal whether the strains isolated in our study have the potential for producing PK and NRP natural products. In addition, the whole-genome sequences combined with LC-MS and Global Natural Products (GNPS) Social Molecular Networking analyses could help reveal the interconnections among metabolites in these actinobacteria.

A previous study [113] analyzed the mechanism of *Streptomyces* strains that could inhibit *E. coli* BW25113, *Pseudomonas aeruginosa* PA01, and *Bacillus subtilis* 168 through a combination of UHPLC-HRMS experiments and genome analysis. They identified that the most common gene clusters for secondary metabolites were those for producing the terpenes. In addition, the production of terpenes by endophytic *Streptomyces* was correlated to the quantities of terpenes within those bryophytes. Terpenoids made by bryophytes are responsible for diverse biological and ecological processes, particularly as defenses against biotic and abiotic stresses [114].

Volatile organic compounds produced by microbes have attracted significant attention for understanding the interactions among organisms, including between plants and microbes. In our analyses of the three strains, many VOCs were identified. Among these, dihydroergotamine and Tris(2,4-di-tert-butylphenyl) phosphate have been reported to be produced by actinobacterium *Exiguobacterium mexicanum* MSSRFS9 [43] that had potent antimicrobial and anti-inflammatory activities, respectively [51,115]. Myristic acid, decane, tetradecane, hexadecane, nonadecane, and heneicosane detected here have shown antimicrobial effects, as demonstrated in previous studies [36,68,69]. Interestingly, many of the VOCs identified here have been detected in plant extracts. For example, extracts of the milky mangrove *Excoecaria agallocha* leaves contained 2.65% of pentadecanoic acid and showed antimicrobial activity against *E. coli*, *S. aureus*, *P. aeruginosa*, and *C. albicans* [97]. Decane was reported in *Artimisia* plants, which is well-known for its anti-malaria activity [31]. The essential oil of *Artimisia aucheri* that can inhibit *E. coli*, *S. aureus*, and *Listeria monocytogenes*, contained decane and other compounds, such as ρ-cymene, 1,8-cineole, and linalool [31]. Endophytic actinobacteria of tropical plants commonly contain NRPS genes and genes of PKS I and PKS II [116]. It is highly likely that the actinobacteria from these plants contributed to the productions of the detected antimicrobial VOCs.

Our study detected significant differences in VOC compositions between the pre-derivatization and post-derivatization samples for each of the three strains. The detected VOC differences were due to differences in how VOCs were generated and prepared for GC-MS analyses by the two sample treatment methods. Specifically, the VOCs detected from the samples before derivatization were those emitted directly by the microbial cultures, while VOCs detected from samples after derivatization include both the originally emitted VOCs and those that were originally not volatile but became volatile after derivatization. In addition, several novel VOCs were detected. Further investigations of these VOCs may allow us to identify their unique structural and functional properties.

Until recently, few studies have focused on volatile organic compounds from actinobacteria. However, a recent study [117] revealed a new role for actinobacterial volatile compounds in modulating nutrient availability and microbial community behavior. The volatile compounds, trimethylamine (TMA), from *Streptomyces venezuelae* influenced a new form of *Streptomyces* growth called “exploration”. TMA can also alter the environment around the exploring area by altering iron availability and enhancing siderophore production and uptake. In our GC-MS analyses of VOCs from the three strains, TMA was not detected. However, this was likely due to the liquid culture medium we used in our study instead of solid agar media needed to show the exploration phenotype. Changing the culture condition could lead to the detection of TMA, other VOCs, and other metabolites.

## 4. Materials and Methods

### 4.1. Sample Collection

Moss samples were collected from Doi Inthanon National Park, Chiang Mai, Thailand, using sterile forceps and stored in zip-lock bags at 4 °C. Mosses were identified to genus and species based on their morphology and microscopical anatomy using the related keys and checklists described in previous publications [118,119,120,121,122,123,124,125,126,127].

### 4.2. Isolation of Actinobacteria from Mosses and Moss-Associated Soils

Two common methods for isolating actinobacteria from natural environments were used in this study to isolate actinobacteria from mosses and moss-associated soils from Thailand. These two methods are described below.

In Method 1, the mosses were first transferred to a sterile 50 mL centrifuge tube and shaken to separate the mosses from the soil attached to them. Soil samples were placed in the silica gel chamber for 3 days and were then used to prepare a serial dilution with Winogradsky’s salts solution (3.8 g K_2_HPO_4_, 1.2 g KH_2_PO_4_, 5.1 g MgSO_4_·7H_2_O, 2.5 g NaCl, 0.05 g Fe_2_(SO_4_)_3_, and 0.05 g MnSO_4_·5H_2_O in 1000 mL deionized water) [128] before plating. The moss samples were rinsed twice with 30 mL sterile distilled water. This was then followed by submerging the moss samples in 1% NaClO for 1 min, 70% EtOH for 1 min, and sterile distilled water for 1 min 2 times. Moss samples were then placed onto sterile filter paper in a silica gel chamber for 3 days before they were weighed and grounded using a sterile mortar. Winogradsky’s salts solution was used to make a serial dilution. Appropriate dilutions of moss samples in Winogradsky’s salts solution were inoculated in water proline agar (1% L-proline) and V8 juice agar (20 mL of V8 Juice, 3 g of CaCO_3_, and 15 g of agar in 1000 mL tap water, pH 7.2) using the pour plate technique. Both media were supplemented with 25 µg/mL nalidixic acid and 25 µg/mL benomyl. Plates from the soil samples and from crushed moss tissues were incubated at 25 °C for 1 week and 4 weeks, respectively. The final rinse water from the surface sterilization step of each moss sample was plated in water proline agar to confirm the effectiveness of the surface sterilization method.

In Method 2, the moss samples were transferred to a sterile 50 mL centrifuge tube and shaken to separate the soil attached to them. The soil samples were dried in a 60 °C oven for 2 h, which were then used to prepare a serial dilution with Winogradsky’s salts solution before plating. For moss samples, they were surface sterilized by submerging in 1% NaClO for 1 min, 70% EtOH for 1 min, and sterile distilled water for 1 min. This step was repeated twice. The surface-sterilized samples were then dried in a 60 °C oven for 2 h before they were weighed and ground using a sterile mortar. The samples were serially diluted in Winogradsky’s salts solution and plated in the same media and incubation condition as described in Method 1.

Presumptive actinobacterial colonies that appeared on isolation plates were picked and purified on Seino’s agar. Seino’s and Waksman agar slants [84] were used to examine the morphology of putative actinobacteria based on their aerial mycelial and substrate mycelial colors. The ISCC-NBS color chart [129] was used to determine the color of mycelia. Actinobacterial isolates were assigned a code with the letter S or P; S indicated the isolates obtained from soil samples, and P indicated the isolates obtained from plant tissue samples.

### 4.3. In Vitro Quantifications of Metabolites with Plant Growth Promoting Potentials

The potential abilities of actinobacteria to produce the following three types of known plant-growth-promoting substances were investigated in this study: indole-3-acetic acid (IAA), siderophores, and tricalcium phosphate solubilizers.

IAA production was determined following the method described previously [130]. In brief, actinobacteria were grown in an ISP2 liquid medium supplemented with 2 mg/mL L-tryptophan for 7 days at 28 °C (dark condition). After 7 days of incubation, the liquid culture was centrifuged at 13,000 rpm, 1 mL of supernatant was mixed with Salkowski’s reagent, and the absorbance at 530 nm was determined.

To determine the production of siderophores, we followed a previously described method [131] for the initial screening. Briefly, Chrome Azurol S (CAS) agar was prepared using King’s B medium as a basal medium [132]. After inoculation, the plates were incubated at 28 °C for 7 days, and the presence of an orange/yellow halozone was used as an indicator of siderophore production. To quantify siderophore production, actinobacteria were further grown in King’s B liquid medium for 7 days at 28 °C (140 rpm) and centrifuged at 13,000 rpm to collect the supernatant. For hydroxamate siderophore production, the method of [133] was followed using desferrioxaminemesylate as standard. In brief, 0.5 mL of supernatant was mixed with 2.5 mL ferric perchlorate in the test tubes and incubated for 5 min. The absorbance was measured at 480 nm. Catecholate siderophore was determined by mixing the supernatant with 0.5 M HCl 1 mL, Nitrite-molybdate reagent 1 mL, and 1 M NaOH 1 mL. The mixture was incubated at room temperature for 5 min, and the absorbance at 500 nm was determined using 2,3-dihydroxybenzoic acid as standard [134].

Phosphate solubilization ability was determined based on the presence and size of a clear zone on Pikovskaya (PVK) agar supplemented with 0.5% tricalcium phosphate [135].

### 4.4. Evaluation of Antimicrobial Activities

The agar plug assay [136] was used to evaluate the antimicrobial activities of actinobacteria from our moss samples. Specifically, a total of seven pathogenic microbial strains were used, including five bacterial pathogens and two fungal pathogens (one fungal pathogen was a yeast and the other was a filamentous fungus). The five bacterial pathogens were: (i) *E. coli* 15-318 (a multidrug-resistant (MDR) strain, resistant to a large number of drugs, such as gentamicin, ampicillin, and tetracycline. The full information of its drug susceptibility was reported by [137]); (ii) *S. aureus* ATCC 8923 (a wild-type, drug-susceptible strain); (iii) a methicillin-resistant *S. aureus* (MRSA)-43300; (iv) *P. syringae* pv. *syringae* 19874; and (v) *P. aeruginosa* 13. The human yeast pathogen was C. albicans 012. For each of these five bacterial and one yeast pathogen, 10^8^ cells were individually spread on the agar surface in a Nunc^®^ Bioassay plate (24.5 × 24.5 × 2.5 cm). Nutrient agar was used for bacterial pathogens and potato dextrose agar was used for *C. albicans*. Pure cultures of actinobacteria on Seino’s Agar were incubated at 28 °C for 30 days prior to the assay. An agar block of each actinobacteria isolate was prepared using a sterile spatula to cut the pure colony of actinobacteria in the size of 1 × 1 cm then placed on the surface of the pathogen-seeded Nunc^®^ Bioassay plate. The plate was incubated overnight, and the potential zone of inhibition was recorded. A sterile blank paper disk (6 mm) loaded with 20 µL distilled water was used as a negative control. In addition, paper disks loaded with 20 µL 10% NaClO and 20 µL 4.25% H_2_O_2_, and BD BBL™ Sensi-Disc™ of 10 µg Ampicillin, and 30 µg tetracycline were used for the evaluation/confirmation of chemical and antibiotic resistance of strains of bacterial pathogens.

To evaluate the activities of actinobacterial strains against the filamentous fungal pathogen *P. destructans*, 10^8^ spores/mL of P. destructans strain US15 (200 µL) were spread on the surface of Sabouraud Dextrose Agar in a Nunc^®^ Bioassay plate. Pure cultures of actinobacteria on Seino’s Agar were incubated at 28 °C for 30 days prior to the assay. An agar block of actinobacteria was prepared using a sterile needle to cut the pure colony of actinobacteria to the size of 1 × 1 cm and placed on the surface of the Nunc^®^ Bioassay plate. The bioassay plate was incubated at 15 °C and the mycelial growth was observed after 14 days. A sterile blank paper disk loaded with 20 µL distilled water was used as a negative control. Paper disks loaded with 20 µL 10% NaClO, 20 µL 4.25% H_2_O_2_, and 20 µL 70% EtOH were used for the determination of the chemical resistance of P. destructans.

The type of inhibition was determined following the criteria of [48]. Specifically, full inhibition against a pathogen means the complete inhibition with no microbial growth within a notable size zone of inhibition. In contrast, partial inhibition has weaker inhibition with some microbial growth in a relatively small zone of inhibition.

The pathogen stock cultures are maintained in the Cheeptham’s Laboratory, Department of Biological Sciences, Thompson Rivers University, Canada.

### 4.5. Identification of Actinobacteria from Mosses

Actinobacteria were grouped according to the morphology and mycelia color that appeared on ISP2 and ISP3 agar [138]. Whole-cell polymerase chain reaction (PCR) was performed using GoTaq^®^ Green Master Mix (Promega) following the manufacturer’s instructions. 8F (5’ AGAGTTTGATCCTGGCTCAG 3’) and 1492R (5’ GGTTACCTTGTTACGACTT 3’) universal primers were used to amplify the 16S rRNA gene. PCR cycling condition was 95 °C for 5 min, 35X (95 °C for 30 s, 55 °C for 30 s, 72 °C for 1.30 min), 72 °C for 5 min. 16S rRNA sequencing was performed at the MOBIX lab at McMaster University, Canada. A sequence comparison of 16S rRNA genes was achieved using the EzBiocloud database (https://www.ezbiocloud.net/) (accessed on 30 November 2020). Sequence trimming and alignment were performed using the BioEdit Sequence Alignment Editor [139]. The neighbor-joining phylogenetic tree was constructed by MEGA7 [140] with 1000 bootstrap replications.

### 4.6. GC-MS Analysis of Metabolites

We selected three strains that showed significant antimicrobial activities for GC-MS analyses of their metabolites. To profile their secreted metabolites, we first grew these three strains (S32-76, S32-77, and S32-79) in Medium 400 [141] at 28 °C for 5 days at 140 rpm. The cells were precipitated through centrifugation. The crude culture supernatant (50 mL) of each isolate was taken and extracted three times with ethyl acetate at a 1:1 (*v*/*v*) ratio. This mixture was shaken vigorously in a separating funnel, and the ethyl acetate layer was collected and evaporated in vacuo to dryness. The dried extract was dissolved in ethyl acetate (10 mg/mL) then transferred into a glass vial. Half of each sample was analyzed directly using GC-MS as described below. The other half was first derivatized before GC-MS analyses. For derivatization, 40 µL of methoxyamination reagent (methoxyamine hydrochloride in pyridine at 20 mg/mL) was added to each extract (~3 mg of dried extract). This mixture was vortexed and incubated at 37 °C, and after 2 h, 70 µL of N-Methyl-N-(trimethylsilyl)trifluoroacetamide (MSTFA) reagent was added. The mixture was vortexed again and incubated at 37 °C for another 30 min, and then centrifuged at 2000 rpm (376 rcf) for 15 min. The supernatant was transferred into a glass vial. The GC-MS profiles were analyzed using a GCMS–QP2020 NX (Shimadzu Co., Japan) equipped with an SH-Rxi-5Sil MS column (0.25 µm df × 0.25 mm ID × 30 m length). Helium (99.9%) was used as the carrier gas at a flow rate of 1 mL/min. The sample was injected at a volume of 1 µL using a split mode with a split ratio of 1:20. The ion source and interface temperature of the mass spectrometer, as well as the injector temperature, were maintained at 250 °C. Mass spectra were obtained by electron ionization (EI) at 70 eV, using a mass scan range of m/z 45-700, with a scan speed of 2500. Detector gain and event time were 0.78 + 0.00 kV and 0.3 s, respectively. The column oven temperature program was set as follows: started at 60 °C (held for 3 min) and raised at a rate of 8 °C/min to 280 °C (held for 25 min). The compounds were identified by matching with the mass spectra in the NIST 17 database. Compounds having spectra that are more than 80% similar to those in the database were annotated.

### 4.7. Bioassay of Secreted Extracellular Compounds against MRSA

To analyze the potential antimicrobial activities of the secreted extracellular compounds, we choose the same three strains mentioned above (32-76, 32-77, and 32-79) that showed strong antimicrobial activities during plate assay. The three selected Streptomyces strains were subjected to small-scale fermentation by being inoculated in a 250 mL flask containing 100 mL of Medium 400. The strains were grown in shake flask conditions at 140 rpm at 28 °C for 5 days. Compound extraction methods were adapted from [142]. A total of 200 mL of cell-free supernatant from each strain was extracted with an equal volume of ethyl acetate for three times using a separation funnel. The crude extracts were dried by a rotary evaporator at 40 °C until they reached half of their volume. Then, the crude extracts were dried completely in a chemical hood. After the drying process, the crude extracts (20 mg) were dissolved in 500 µL absolute ethanol. For testing the potential antimicrobial activity of crude extracts, 40 µL of crude extract from each strain was loaded onto a sterile 6 mm paper disk; we then waited until the disk was completely dry before placing it onto nutrient agar overlayed with an MRSA cell lawn. A paper disk loaded with 40 µL absolute ethanol and then dried by air was used as a negative control.

### 4.8. Statistical Analysis

Statistical analysis of clear zones on PVK agar was performed by SPSS statistics 17.0 program.

## 5. Conclusions

In this study, the species of moss, culture medium, and pre-treatment condition all influenced the isolation of actinobacteria from mosses and from moss-associated rhizospheric soil. Our analyses revealed diverse actinobacteria associated with mosses in several genera, *Streptomyces*, *Micromonospora*, *Nocardia*, *Actinoplanes*, *Saccharothrix*, *Streptosporangium*, and *Cryptosporangium*. Many of these actinobacteria can produce IAA, siderophores, enzymes to solubilize tricalcium phosphate, antimicrobial compounds effective against plant pathogens, and signaling molecules for potential communication with other species. Indeed, GC-MS analyses indicated that several *Streptomyces* strains can produce a large number of bioactive compounds, including antimicrobials. Importantly, the actinobacteria from mosses can inhibit the growth of plant, human, and bat pathogens, including multidrug-resistant strains, demonstrating the significant potential for the discovery of novel antimicrobial agents. Together, our research suggests a number of avenues from which to further explore the roles of actinobacteria in drug discovery and in microbe–microbe and plant–microbe interactions [143].

## Figures and Tables

**Table 1:** Actinobacteria obtained from mosses using two different methods. Method 1 used a pre-treatment temperature of 25 °C; method 2 used a pre-treatment temperature of 60 °C.

Method	Host Moss Species	Location	Isolation Media	Average Value CFU/g		No. Isolates
				Rhizosphere Soil	Inside Moss	
Method 1	*P. microstomum*	8°35′19′’ N98°29′12′’ E	WPA	1.3 × 10^4^	0	15
			V8 juice agar	4.6 × 10^4^	0	36
	*Hypnum* sp.	8°35′19′’ N98°29′12′’ E	WPA	5.9 × 10^4^	0	23
			V8 juice agar	9.5 × 10^3^	0	6
	*P. maximoviczii*	8°35′19′’ N98°29′12′’ E	WPA	1.5 × 10^3^	2 × 10^3^	3
			V8 juice agar	4.4 × 10^4^	2 × 10^3^	0
Method 2	*B. buchananii*	18°33′14′’ N98°46′52′’ E	WPA	1.1 × 10^4^	4.5 × 10^3^	30
			V8 juice agar	7.5 × 10^3^	0	45
	*D. maschalogena*	18°40′44′’ N98°50′25′’ E	WPA	0	2.9 × 10^5^	0
			V8 juice agar	0	3 × 10^5^	2
	*B. recurvulum*	18°40′44′’ N98°50′25′’ E	WPA	0	3.5 × 10^4^	11
			V8 juice agar	5 × 10^3^	2.3 × 10^4^	14
	*T. cymbifolium*	8°35′19′’ N98°29′12′’ E	WPA	1.1 × 10^4^	0	2
			V8 juice agar	7.5 × 10^3^	0	0
	*M. submacrocarpum*	8°35′19′’ N98°29′12′’ E	WPA	0	4.9 × 10^4^	4
			V8 juice agar	0	6.7 × 10^4^	5
	*C. purpureus*	18°40′44′’ N98°50′25′’ E	WPA	0	0	0
			V8 juice agar	5 × 10^3^	0	0
Total						196

**Table 2:** Actinobacterial isolates capable of phosphate solubilization. Data for each isolate is based on three repeats (*n* = 3). ^a–d^: isolates with different letters indicate statistically significant differences as determined by SPSS one-way ANOVA and Tukey HSD test (*p* < 0.05).

Source	Host Moss Species	Isolate	Clear Zone on PVK Agar (cm)
Plant tissue	*B. buchananii*	P32-20	0.71 ± 0.11 ^ab^
	*D. maschalogena*	P33-17	0.96 ± 0.13 ^ab^
	*M. submacrocarpum*	P49-10	0.71 ± 0.05 ^ab^
	*M. submacrocarpum*	P49-11	0.85 ± 0.08 ^ab^
	*M. submacrocarpum*	P49-14	1.21 ± 0.74 ^bc^
Soil	*P. microstomum*	S3-11	0.86 ± 0.06 ^ab^
	*P. microstomum*	S3-16	0.95 ± 0.01 ^ab^
	*P. microstomum*	S3-17	0.62 ± 0.03 ^ab^
	*P. microstomum*	S3-31	0.54 ± 0.05 ^a^
	*Hypnum* sp.	S6-3	1.04 ± 0.04 ^ab^
	*Hypnum* sp.	S6-6	0.63 ± 0.04 ^ab^
	*Hypnum* sp.	S6-14	0.51 ± 0.33 ^a^
	*Hypnum* sp.	S6-17	0.6 ± 0 ^ab^
	*Hypnum* sp.	S6-28	1.75 ± 0.12 ^d^
	*Hypnum* sp.	S6-31	1.36 ± 0.08 ^ab^
	*P. maximoviczii*	S13-2	0.75 ± 0.14 ^ab^
	*B. buchananii*	S32-30	0.77 ± 0.21 ^ab^
	*B. buchananii*	S54-2	0.91 ± 0.02 ^ab^
	*B. buchananii*	S54-18	0.92 ± 0.1 ^ab^
	*B. buchananii*	S54-19	0.87 ± 0.06 ^ab^

**Table 3:** 16S rRNA sequencing results of actinobacteria from mosses and comparisons with those in the EzBiocloud database.

Genera	Isolate	DDBJ Accession Number	Top Hit Taxon	% Similarity	Length (bp)
*Streptomyces*	P32-2	LC551864	*Streptomyces althioticus* NRRL B-3981^T^	100	1221
	P32-15	LC551863	*Streptomyces althioticus* NRRL B-3981^T^	100	1221
	P32-21	LC551860	*Streptomyces althioticus* NRRL B-3981^T^	100	1221
	P54-7	LC551871	*Streptomyces althioticus* NRRL B-3981^T^	100	1328
	P54-15	LC551870	*Streptomyces althioticus* NRRL B-3981^T^	100	1329
	P49-13	LC551869	*Streptomyces violaceolatus* DSM 40438^T^	100	1344
	P49-18	LC551868	*Streptomyces griseoincarnatus* LMG 19316^T^	100	1303
	S3-11	LC551876	*Streptomyces rhizosphaerihabitans* JR-35^T^	99.85	1324
	S3-16	LC551877	*Streptomyces mirabilis* NBRC 13450^T^	100	1338
	S3-17	LC551878	*Streptomyces aureus* NBRC 100912^T^	100	1355
	S3-26	LC551872	*Streptomyces wedmorensis* NRRL 3426^T^	99.55	1324
	S3-30	LC551874	*Streptomyces camponoticapitis* 2H-TWYE14^T^	99.62	1329
	S6-17	LC551893	*Streptomyces setonii* NRRL ISP-5322^T^	100	1329
	S32-79	LC551890	*Streptomyces setonii* NRRL ISP-5322^T^	100	1335
	S6-31	LC551895	*Streptomyces sporoverrucosus* NBRC 15458^T^	100	1360
	S32-5	LC551886	*Streptomyces fulvissimus* DSM 40593^T^	99.93	1359
	S32-27	LC551891	*Streptomyces fulvissimus* DSM 40593^T^	99.93	1360
	S32-55	LC551884	*Streptomyces fulvissimus* DSM 40593^T^	99.93	1356
	S32-76	LC551888	*Streptomyces fulvissimus* DSM 40593^T^	99.93	1353
	S32-10	LC551885	*Streptosporangium oxazolinicum* K07-0460^T^	99.25	1343
	S32-29	LC551887	*Streptomyces badius* NRRL B-2567^T^	100	1300
	S32-43	LC551880	*Streptomyces badius* NRRL B-2567^T^	100	1329
	S32-52	LC551882	*Streptomyces omiyaensis* NBRC 13449^T^	99.55	1343
	S32-65	LC551889	*Streptomyces globisporus* NBRC 12867^T^	100	1337
	S32-74	LC551883	*Streptomyces wedmorensis* NRRL 3426^T^	99.62	1330
	S32-77	LC551881	*Streptomyces dioscori* A217^T^	99.63	1341
*Micromonospora*	P32-13	LC551861	*Micromonospora maritima* D10-9-5^T^	99.77	1324
	P32-19	LC551862	*Micromonospora marina* DSM 45555^T^	99.85	1331
	P33-11	LC551867	*Micromonospora aurantiaca* ATCC 27029^T^	100	1296
	S32-33	LC551892	*Micromonospora tulbaghiae* DSM 45142^T^	100	1326
*Actinoplanes*	S3-33	LC551879	*Actinoplanes lutulentus* NEAU-GRX6^T^	98.57	1327
	S6-21	LC551896	*Actinoplanes consettensis* JCM 7624^T^	99.93	1338
*Saccharothrix*	S3-21	LC551875	*Saccharothrix yanglingensis* Hhs.015^T^	98.86	1328
	S3-31	LC551873	*Saccharothrix yanglingensis* Hhs.015^T^	98.86	1332
*Nocardia*	P33-17	LC551866	*Nocardia fluminea* S1^T^	99.55	1332
	S6-27	LC551894	*Nocardia salmonicida* subsp. *cummidelens* R89^T^	100	1335
*Cryptosporangium*	P33-8	LC551865	*Cryptosporangium minutisporangium* IFO 15962^T^	99.92	1330

**Table 4:** Major classes of compounds detected by GC-MS before and after derivatization.

Condition	Compound Nature	Estimated Compound Content (%)
Pre-derivatization	Alkane	46.75
	Alcohol	12.99
	Ketone	3.90
	Carboxylic acid	2.60
	Flavonoid	2.60
	Amide	2.60
	Alkaloid derivative	2.60
	Ether	2.60
	Imide	1.30
	Ester	1.30
	Lactone	1.30
	Others	19.48
Post-derivatization	Alkanes	15.29
	Carboxylic acid	10.59
	Sugar	8.24
	Alcohol	7.06
	Sugar alcohol	5.88
	Fatty acid	4.71
	Ether	3.53
	Ester	2.35
	Amide	2.35
	Imide	2.35
	Terpene	2.35
	Others	30.59

**Table 5:** The anti-MRSA activity measured after incubation for 3 days at 28 °C on nutrient agar. The zone of inhibition indicated activity against the growth of MRSA. Each disk contained 1.6 mg of crude extract.

Isolates	Activity of Crude Extract against MRSA (mm, ± Standard Error)
Absolute Ethanol (negative control)	0 (±0)
Tetracycline (positive control)	8 (±0.3)
S32-76	10 (±0.3)
S32-77	13 (±0.4)
S32-79	14 (±0.4)

## Data Availability

Accession numbers for all 37 16S rRNA sequences used in this study can be found listed in Table 3.

## Supplementary Materials

The following are available online at https://www.mdpi.com/article/10.3390/metabo12010022/s1, Figure S1: GC-MS chromatogram of metabolites produced from actinobacteria; Table S1: Bioactive compounds identified by GC-MS of *Streptomyces* spp. ethyl acetate extract.
