# Supplementary material for: Taxonomic and Metabolite Diversities of Moss-Associated Actinobacteria from Thailand
Source: Metabolites. 2021 Dec 27;12(1):22. doi: 10.3390/metabo12010022 (PMC8777641; doi:10.3390/metabo12010022)
Supplement: Supplementary file 1 [file metabolites-12-00022-s001.zip › Supplementary Figure S1.pdf]

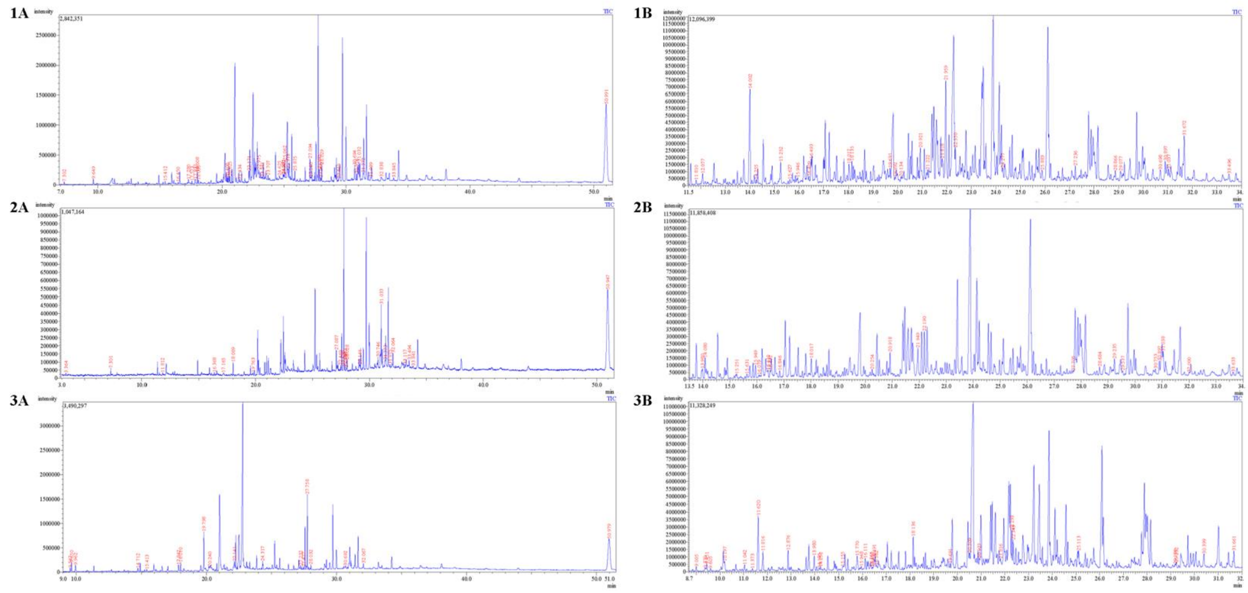

Supplementary Figure S1. GC-MS chromatogram of metabolites produced from actinobacteria. A: Pre-derivatization. B: Post-derivatization. Strain 1: S32-76. Strain 2: S32-77. Strain 3: S32-79.
