# Supplementary material for: Taxonomic and Metabolite Diversities of Moss-Associated Actinobacteria from Thailand
Source: Metabolites. 2021 Dec 27;12(1):22. doi: 10.3390/metabo12010022 (PMC8777641; doi:10.3390/metabo12010022)
Supplement: Supplementary file 1 [file metabolites-12-00022-s001.zip › Supplementary Table S1.pdf]

Condition	Isolate	No.	Retention time	Compound	Peak area %	Molecular weight	Formula	Reported activity	References
Underivatized	S32-76	1	7.302	Decane	0.09	142	$C_{10}H_{22}$	Antimicrobial	[31]
		2	9.649	Phenylethyl Alcohol	0.2	122	$C_8H_{10}O$	Antimicrobial	[32]
		3	15.412	1,1'-(1,3-phenylene)bis-ethanone	0.1	162	$C_{10}H_{10}O_2$	No reported antimicrobial	This study
		4	16.45	Tetraglyme	0.07	222	$C_{10}H_{22}O_5$	No reported antimicrobial	This study
		5	17.28	5-Methyl-tetradecane	0.1	212	$C_{15}H_{32}$	No reported antimicrobial	This study
		6	17.573	3-Methyl-pentadecane	0.08	226	$C_{16}H_{34}$	No reported antimicrobial	This study
		7	17.837	3,6-Diisopropyl-2,5-dioxomorpholine	0.22	199	$C_{10}H_{17}NO_3$	Antioxidant and anticancer	[33]
		8	18.008	Hexadecane	0.35	226	$C_{16}H_{34}$	Antibacterial	[34]
		9	18.095	Cyclotetradecane	0.15	196	$C_{14}H_{28}$	Antimicrobial	[35]
		10	20.408	3-Methyl-heptadecane	0.18	254	$C_{18}H_{38}$	Antimicrobial	[36]
		11	20.625	Palustrol	0.23	222	$C_{15}H_{26}O$	Antifungal, Antioxidant	[37]
		12	21.434	Octyl-.beta.-D-glucopyranoside	0.1	292	$C_{14}H_{28}O_6$	Antimicrobial	[38]
		13	22.171	7,9-Di-tert-butyl-1-oxaspiro(4,5)deca-6,9-diene-2,8-dione	0.37	276	$C_{17}H_{24}O_3$	No reported antimicrobial	This study
		14	22.975	2-Methyl-eicosane	0.56	296	$C_{21}H_{44}$	Antibacterial	[39]
		15	23.241	1-Propyltridecyl 4-cyanobenzoate	0.25	371	$C_{24}H_{37}NO_2$	Antimicrobial	This study
		16	23.527	Octylcyclodecane	0.3	252	$C_{18}H_{36}$	No reported antimicrobial	This study
		17	23.707	Humulenol-II	0.15	220	$C_{15}H_{24}O$	Antibacterial, Antifungal	[40]
		18	24.625	Indole-3-glyoxylic acid methyl ester	0.13	203	$C_{11}H_9NO_3$	No reported antimicrobial	This study
		19	24.931	9-Methylheneicosane	0.19	310	$C_{22}H_{46}$	No reported antimicrobial	This study
		20	24.982	Pentacosane	0.19	352	$C_{25}H_{52}$	Antioxidant, Antimirobial	[41-42]
		21	25.062	5-Methyl-heneicosane	0.75	310	$C_{22}H_{46}$	No reported antimicrobial	This study
		22	25.318	3-Methyl-heneicosane	0.35	310	$C_{22}H_{46}$	No reported antimicrobial	This study

	23	25.875	4-Cyclohexyl-decane	0.34	224	$C_{16}H_{32}$	Antimicrobial	[36]
	24	27.094	Dihydroergotamine	0.83	583	$C_{33}H_{37}N_5O_5$	Antibacterial	[43]
	25	27.14	Sulfurous acid, 2-ethylhexyl octadecyl ester	0.26	446	$C_{26}H_{54}O_3S$	No reported antimicrobial	This study
	26	27.845	Pinostrobin	0.45	270	$C_{16}H_{14}O_4$	Antimicrobial	[44]
	27	28.029	Octadecylcyclohexane	0.62	336	$C_{24}H_{48}$	Antimicrobial	[45]
	28	29.41	1,3-Propanediol, ethyl tetracosyl ether	0.08	440	$C_{29}H_{60}O_2$	No reported antimicrobial	This study
	29	30.694	Tetracontane-1,40-diol	0.63	594	$C_{40}H_{82}O_2$	Antibacterial	[46]
	30	30.856	(Z)-14-Methyl-hexadec-8-enal	0.22	252	$C_{17}H_{32}O$	Antimicrobial	[47]
	31	31.032	10,11-Dihydrofarnesol	0.83	224	$C_{15}H_{28}O$	No reported antimicrobial	This study
	32	31.372	3-Methyl-octadecane	0.19	268	$C_{19}H_{40}$	No reported antimicrobial	This study
	33	31.999	Octacosanol	0.13	410	$C_{28}H_{58}O$	Anti-Oxidant and Anti-Bacteria	[48]
	34	32.838	Tetrapentacontane	0.33	758	$C_{54}H_{110}$	Antioxidant and Antimicrobial	[49-50]
	35	33.845	1-Iodo-octacosane	0.11	520	$C_{28}H_{57}I$	No reported antimicrobial	This study
	36	50.991	Tris(2,4-di-tert-butylphenyl) phosphate	14.31	662	$C_{24}H_{63}O_4P$	Anti-inflammatory	[51]
S32-77	1	3.364	Octane	0.11	114	C_8H_{18}	No reported antimicrobial	This study
	2	7.301	Decane	0.28	142	$C_{10}H_{22}$	Antimicrobial	[31]
	3	11.812	3-Methyl-N-isobutylbutyramide	0.11	157	$C_9H_{19}NO$	No reported antimicrobial	This study
	4	16.368	β,2,3-trimethyl-cyclopentaneethanol	0.11	156	$C_{10}H_{20}O$	No reported antimicrobial	This study
	5	17.165	3-Methyl-piperazine-2,5-dione	0.27	128	$C_5H_8N_2O_2$	No reported antimicrobial	This study
	6	18.009	Hexadecane	0.5	226	$C_{16}H_{34}$	Antibacterial	This study
	7	19.763	DL-Alanyl-l-leucine	0.12	202	$C_9H_{18}N_2O_3$	No reported antimicrobial	[33]
	8	27.087	Dihydroergotamine	0.95	583	$C_{33}H_{37}N_5O_5$	Antibacterial compound	This study

	9	27.469	1-Iodo-docosane	0.25	436	$C_{22}H_{45}I$	No reported antimicrobial	[43]
	10	27.699	Octacosanol	0.25	410	$C_{28}H_{58}O$	Anti-Oxidant and Anti-bacterial	This study
	11	27.842	Pinostrobin	0.38	270	$C_{16}H_{14}O_4$	Antimicrobial	[48]
	12	27.966	1-Octadecanol, methyl ether	0.21	284	$C_{19}H_{40}O$	Antifungal	[44]
	13	28.028	Octadecylcyclohexane	0.6	336	$C_{24}H_{48}$	Antimicrobial	[52]
	14	29.144	7-Methyl-hexadecane	0.39	240	$C_{17}H_{36}$	No reported antimicrobial	[45]
	15	30.746	10,11-Dihydrofarnesol	0.54	224	$C_{15}H_{28}O$	No reported antimicrobial	This study
	16	31.033	Crotonic acid, menthyl ester	3.17	224	$C_{14}H_{24}$O2	Anticandidal and anti-carcinogenic	This study
	17	31.373	3-Methyl-octadecane	0.3	268	$C_{19}H_{40}$	No reported antimicrobial	[53]
	18	31.815	2,6,10,14,18-Pentamethyl-2,6,10,14,18-eicosapentaene	0.14	342	$C_{25}H_{42}$	No reported antimicrobial	This study
	19	32.064	4-cyclohexyl-Decane	0.88	224	$C_{16}H_{32}$	Antimicrobial	This study
	20	33.117	2-Dodecen-1-yl(-)succinic anhydride	0.22	266	$C_{16}H_{26}O_3$	Antimicrobial, Antioxidant	[36]
	21	33.494	5-Methyl-heneicosane	0.39	310	$C_{22}H_{46}$	No reported antimicrobial	[54]
	22	33.841	1-Iodo-octacosane	0.15	520	$C_{28}H_{57}I$	No reported antimicrobial	This study
	23	50.947	Tris(2,4-di-tert-butylphenyl) phosphate	18.13	662	$C_{42}H_{63}O_4P$	Anti-inflammatory	This study
	1	9.547	Maltol	0.24	126	$C_6H_6O_3$	Antibacterial, Antioxidant	[51]
	2	9.65	Phenylethyl alcohol	0.52	122	$C_8H_{10}O$	Antimicrobial	[55]
	3	9.962	Succinimide	0.53	99	$C_4H_5NO_2$	No reported antimicrobial	[32]
S32-79	4	14.712	N-Hydroxymethyl-2-phenylacetamide	0.26	165	$C_9H_{11}NO_2$	No reported antimicrobial	This study
	5	15.413	1,1'-(1,3-Phenylene)bis-ethanone	0.11	162	$C_{10}H_{10}O_2$	No reported antimicrobial	This study
	6	17.842	3,6-Diisopropyl-2,5-dioxomorpholine	0.42	199	$C_{10}H_{17}NO_3$	Antioxidant	This study
	7	18.01	Hexadecane	0.45	226	$C_{16}H_{34}$	Antibacterial	[33]

		8	19.798	3,4-diamino-6-methyl-1H-Pyrolo[3,4-c]pyridin-1-one	2.63	176	$C_8H_8N_4O$	No reported antimicrobial	[34]
		9	20.24	Tryptophol	0.23	161	$C_{10}H_{11}NO$	Antibiotic	This study
		10	22.141	2-Amino-6-methyl-pyrido[2,3-d]pyrimidine-4(3H)-one	0.93	176	$C_8H_8N_4O$	No reported antimicrobial	[56]
		11	24.317	n-Heptadecanol-1	0.47	256	$C_{17}H_{36}O$	Antimalarial, Antifungal, Antioxidant	This study
		12	27.232	2,4-Dimethyl-docosane	0.33	338	$C_{24}H_{50}$	Antimicrobial	[57]
		13	27.472	3-Methyl-octadecane	0.12	268	$C_{19}H_{40}$	No reported antimicrobial	[58]
		14	27.758	Pentacosane	4.2	352	$C_{25}H_{52}$	Antioxidant, Antimirobial	This study
		15	28.032	4-Cyclohexyl-undecane	0.29	238	$C_{17}H_{34}$	No reported antimicrobial	[41-42]
		16	30.692	1,54-Dibromo-tetrapentacontane	0.18	914	$C_{54}H_{108}Br_2$	No reported antimicrobial	This study
		17	32.067	4-Cyclohexyl-dodecane	0.37	252	$C_{18}H_{36}$	Antimicrobial, antifungal	This study
		18	50.979	Tris(2,4-di-tert-butylphenyl) phosphate	10.59	662	$C_{42}H_{63}O_4P$	Anti-inflammatory	[36]
Derivatized	S32-76	1	11.81	Phenylethyl Alcohol, TMS derivative	0.06	194	$C_{11}H_{18}OSi$	Antimicrobial	[51]
		2	12.077	Urea, 2TMS derivative	0.19	204	$C_7H_{20}N_2OSi_2$	Bacteriostatic and bactericidal	[32]
		3	14.002	Pyrrole-2-carboxylic acid, 2TMS derivative	1.92	255	$C_{11}H_{21}NO_2Si_2$	No reported antimicrobial	[59]
		4	14.255	p-Toluic acid, TMS derivative	0.06	208	$C_{11}H_{16}O_2Si$	No reported antimicrobial	This study
		5	15.252	1-Phenyl-1,2-ethanediol, 2TMS derivative	0.32	282	$C_{14}H_{26}O_2Si_2$	No reported antimicrobial	This study
		6	15.627	N-(trimethylsilyl)-Melamine	0.07	198	$C_6H_{14}N_6Si$	No reported antimicrobial	This study
		7	15.946	p-Benzoquinone	0.06	220	$C_{14}H_{20}O_2$	No reported antimicrobial	This study
		8	16.409	Erythritol, 4TMS derivative	0.08	410	$C_{16}H_{42}O_4Si_4$	Antimicrobial	This study
		9	16.493	4-Hydroxybenzyl alcohol, 2TMS derivative	0.34	268	$C_{13}H_{24}O_2Si_2$	Antioxidant	[60-61]
		10	18.017	Hexadecane	0.39	226	$C_{16}H_{34}$	Antimicrobial	[62]
		11	18.135	Anthranilic acid, 2TMS derivative	0.36	281	$C_{13}H_{23}NO_2Si_2$	No reported antimicrobial	[63,35]

	12	19.691	Xylitol, 5TMS derivative	0.2	512	$C_{20}H_{52}O_5Si_5$	Antimicrobial	This study
	13	19.892	2,3-Dihydroxybenzoic acid, 3TMS derivative	0.07	370	$C_{16}H_{30}O_4Si_3$	Catecholate siderophore, Antimicrobial	[64-65]
	14	20.134	5-Methyl-tetradecane	0.05	212	$C_{15}H_{32}$	No reported antimicrobial	[18,66-67]
	15	20.921	Myristic acid, TMS derivative	0.52	300	$C_{17}H_{36}O_2Si$	Antimicrobial, antifungal	This study
	16	21.222	Tryptophol, TMS derivative	0.14	233	$C_{13}H_{19}NOSi$	Antibiotics	[68-69]
	17	21.818	(1E)-d-Glucose-2,3,4,5,6-pentakis-O-(trimethylsilyl)-o-methyloxyme	0.5	569	$C_{22}H_{55}NO_6Si_5$	No reported antimicrobial	[56]
	18	21.959	Galactose oxime, 6TMS derivative	2.07	627	$C_{24}H_{61}NO_6Si_6$	Anti-inflammatory	This study
	19	22.35	Glucose, 5TMS derivative	0.44	540	$C_{21}H_{52}O_6Si_5$	No reported antimicrobial	[70]
	20	24.277	(10Z)-Heptadecenoic acid, TMS derivative	0.23	340	$C_{20}H_{40}O_2Si$	No reported antimicrobial	This study
	21	25.883	Octadecyl-cyclohexane	0.21	336	$C_{24}H_{48}$	No reported antimicrobial	This study
	22	27.236	5-Methyl-heneicosane	0.19	310	$C_{22}H_{46}$	No reported antimicrobial	This study
	23	28.866	tert.-butyldimethylsilyl ether-5-Hydroxy-7-methoxyflavanone	0.14	384	$C_{22}H_{28}O_4Si$	No reported antimicrobial	This study
	24	29.077	11-Methylpentacosane	0.21	366	$C_{26}H_{54}$	No reported antimicrobial	This study
	25	30.698	Hexadecyl-oxirane	0.19	268	$C_{18}H_{36}O$	Antimicrobial	This study
	26	30.897	β-Gentiobiose, octakis(trimethylsilyl) ether, methyloxime (isomer 1)	0.36	947	$C_{37}H_{89}NO_{11}Si_8$	No reported antimicrobial	[71]
	27	31.037	(E)-Hexadec-2-enal	0.22	238	$C_{16}H_{30}O$	No reported antimicrobial	This study
	28	31.672	Tetracontane	0.8	562	$C_{40}H_{82}$	Antimicrobial , antioxidant	This study
	29	33.496	5-Methylnonacosane	0.12	422	$C_{30}H_{62}$	No reported antimicrobial	[49]
S32-77	1	13.98	Pyrrole-2-carboxylic acid, 2TMS derivative	0.14	255	$C_{11}H_{21}NO_2Si_2$	Antifungal	This study
	2	14.08	Acetin, bis-1,2-trimethylsilyl ether	0.33	278	$C_{11}H_{26}O_4Si_2$	No reported antimicrobial	[72]

3	15.251	1-Phenyl-1,2-ethanediol, 2TMS derivative	0.08	282	$C_{14}H_{26}O_2Si_2$	No reported antimicrobial	This study
4	15.631	N-(trimethylsilyl)-Melamine	0.08	198	$C_6H_{14}N_6Si$	No reported antimicrobial	This study
5	15.949	p-Benzoquinone	0.23	220	$C_{14}H_{20}O_2$	No reported antimicrobial	This study
6	16.079	2,5-Bis[(trimethylsilyl)oxy]-4H-imidazol-4-one	0.08	258	$C_9H_{18}N_2O_3Si_2$	No reported antimicrobial	This study
7	16.41	Erythritol, 4TMS derivative	0.17	410	$C_{16}H_{42}O_4Si_4$	Antimicrobial	This study
8	16.492	4-Hydroxybenzyl alcohol, 2TMS derivative	0.14	268	$C_{13}H_{24}O_2Si_2$	Antioxidant	[60-61]
9	16.846	Benzenebutanoic acid, TMS derivative	0.1	236	$C_{13}H_{20}O_2Si$	Antimicrobial	[62]
10	18.017	Hexadecane	0.36	226	$C_{16}H_{34}$	Antimicrobial	[73]
11	20.254	1,2,3,5-Tetrakis-O-(trimethylsilyl)-β-D-arabinofuranose	0.12	438	$C_{17}H_{42}O_5Si_4$	Antimicrobial, Anti-inflammatory	[63,35]
12	20.918	Myristic acid, TMS derivative	0.41	300	$C_{17}H_{36}O_2Si$	Antimicrobial, Antifungal	[74]
13	21.949	pentakis(trimethylsilyl) ether-D-Allose, , methyloxime (syn)	0.75	569	$C_{22}H_{55}NO_6Si_5$	No reported antimicrobial	[68-69]
14	22.19	Pentadecanoic acid, TMS derivative	0.91	314	$C_{18}H_{38}O_2Si$	Antimicrobial	This study
15	27.705	Bacteriochlorophyll-c-stearyl	0.07	840	$C_{52}H_{72}MgN_4O_4$	No reported antimicrobial	[69]
16	28.684	(Z)-Docos-9-enenitrile	0.15	319	$C_{22}H_{41}N$	No reported antimicrobial	This study
17	29.235	5-Methyl-heneicosane	0.27	310	$C_{22}H_{46}$	No reported antimicrobial	This study
18	29.537	1-Monopalmitin, 2TMS derivative	0.08	474	$C_{25}H_{54}O_4Si_2$	No reported antimicrobial	This study
19	30.753	3,3'-Bi-p-menthane	0.1	278	$C_{20}H_{38}$	No reported antimicrobial	This study
20	30.897	octakis(trimethylsilyl) ether-β-Gentiobiose, methyloxime (isomer 1)	0.3	947	$C_{37}H_{89}NO_{11}Si_8$	No reported antimicrobial	This study
21	31.038	Crotonic acid, menthyl ester	0.47	224	$C_{14}H_{24}O_2$	Anticandidal and anti-carcinogenic	This study
22	32	1-Methoxyoctacosane	0.07	424	$C_{29}H_{60}O$	No reported antimicrobial	[53]

	23	33.633	L-Tryptophan, 1-(trimethylsilyl)-, trimethylsilyl ester	0.08	348	$C_{17}H_{28}N_2O_2Si_2$	No reported antimicrobial	This study
S32-79	1	9.005	Pyruvic acid, TMS derivative	0.07	160	$C_6H_{12}O_3Si$	Antimicrobial	This study
	2	9.37	tert-Butylpentamethyldisiloxane	0.09	204	$C_9H_{24}OSi_2$	No reported antimicrobial	[75]
	3	9.451	N-(Trimethylsilyl)hexanamide	0.1	187	$C_9H_{21}NOSi$	No reported antimicrobial	This study
	4	9.605	Hydroxylamine, 3TMS derivative	0.04	249	$C_9H_{27}NOSi_3$	No reported antimicrobial	This study
	5	10.197	1-(Trimethylsilyl)-2-piperidinone	0.2	171	$C_8H_{17}NOSi$	No reported antimicrobial	This study
	6	11.042	(3R)-Pantolactone, TMS derivative	0.13	202	$C_9H_{18}O_3Si$	No reported antimicrobial	This study
	7	11.373	2-Methylundecane	0.04	170	$C_{12}H_{26}$	No reported antimicrobial	This study
	8	11.62	Hymexazole, tert-butyldimethylsilyl ether	1.21	213	$C_{10}H_{19}NO_2Si$	No reported antimicrobial	This study
	9	11.816	Phenylethyl Alcohol, TMS derivative	0.39	194	$C_{11}H_{18}OSi$	Antimicrobial	This study
	10	12.876	Maltol, TMS derivative	0.34	198	$C_9H_{14}O_3Si$	Antibacterial, Antioxidant	[32]
	11	13.98	Pyrrole-2-carboxylic acid, 2TMS derivative	0.29	255	$C_{11}H_{21}NO_2Si_2$	No reported antimicrobial	[55]
	12	14.185	1,2,4-Butanetriol, 3TMS derivative	0.06	322	$C_{13}H_{34}O_3Si_3$	No reported antimicrobial	This study
	13	14.252	p-Toluic acid, TMS derivative	0.05	208	$C_{11}H_{16}O_2Si$	No reported antimicrobial	This study
	14	15.155	α-Gurjunene	0.17	204	$C_{15}H_{24}$	No reported antimicrobial	This study
	15	15.77	Benzeneacetamide, TMS derivative	0.41	207	$C_{11}H_{17}NOSi$	Antimicrobial	This study
	16	15.948	p-Benzoquinone	0.04	220	$C_{14}H_{20}O_2$	No reported antimicrobial	[76,77]
	17	16.111	5-Hydroxymaltol, 2-O-TMS	0.21	286	$C_{12}H_{22}O_4Si_2$	Antioxidant	This study
	18	16.365	4-(Methoxycarbonyl)phenol, TMS derivative	0.04	224	$C_{11}H_{16}O_3Si$	No reported antimicrobial	[78]
	19	16.41	Erythritol, 4TMS derivative	0.03	410	$C_{16}H_{42}O_4Si_4$	Antimicrobial	This study
	20	16.491	4-Hydroxybenzyl alcohol, 2TMS derivative	0.21	268	$C_{13}H_{24}O_2Si_2$	Antioxidant	[60-61]
	21	16.582	N-(2-Phenylethyl)-acetamide	0.08	163	$C_{10}H_{13}NO$	No reported antimicrobial	[62]
	22	18.136	Anthranilic acid, 2TMS derivative	0.55	281	$C_{13}H_{23}NO_2Si_2$	No reported antimicrobial	This study

23	19.691	Ribitol, 5TMS derivative	0.06	512	$C_{20}H_{52}O_5Si_5$	No reported antimicrobial	This study
24	20.509	3,5-Di-tert-butyl-2-trimethylsilyloxybenzaldehyde	0.4	306	$C_{18}H_{30}O_2Si$	No reported antimicrobial	This study
25	20.927	Myristic acid, TMS derivative	0.2	300	$C_{17}H_{36}O_2Si$	Antimicrobial, antifungal	This study
26	21.816	(1E)-d-Glucose 2,3,4,5,6-pentakis-O-(trimethylsilyl)-o-methyloxyme	0.27	569	$C_{22}H_{55}NO_6Si_5$	No reported antimicrobial	[68-69]
27	22.293	Pentadecanoic acid, TMS derivative	0.68	314	$C_{18}H_{38}O_2Si$	Antimicrobial	This study
28	22.349	Glucose, 5TMS derivative	0.56	540	$C_{21}H_{52}O_6Si_5$	No reported antimicrobial	[69]
29	25.113	Dimethyl(octadecyloxy) propyl silane	0.32	370	$C_{23}H_{50}OSi$	No reported antimicrobial	This study
30	29.18	1-Monopalmitin, 2TMS derivative	0.07	474	$C_{25}H_{54}O_4Si_2$	No reported antimicrobial	This study
31	29.232	5-Methyl-heneicosane	0.09	310	$C_{22}H_{46}$	No reported antimicrobial	This study
32	30.399	1,2,3,5-Tetrakis-O-(trimethylsilyl)-β-D-arabinofuranose	0.28	438	$C_{17}H_{42}O_5Si_4$	Antimicrobial, anti-inflammatory	This study
33	31.661	Tetracontane	0.33	562	$C_{40}H_{82}$	Antimicrobial, antioxidant	[74]

Table S1. Bioactive compounds identified by GC-MS of Streptomyces spp. ethyl acetate extract.
